# Social media and urology: The good, the bad and the ugly

**DOI:** 10.1177/03915603241273885

**Published:** 2024-08-30

**Authors:** Patrick Juliebø-Jones, Vineet Gauhar, Etienne Xavier Keller, Vincent De Coninck, Ali Talyshinskii, Alba Sierra, Eugenio Ventimiglia, Lazaros Tzelves, Mariela Corrales, Esteban Emiliani, Christian Beisland, Bhaskar K Somani

**Affiliations:** 1 Department of Urology, Haukeland University Hospital, Bergen, Norway; 2 Department of Clinical Medicine, University of Bergen, Bergen, Norway; 3 EAU YAU Urolithiasis Group, Arnhem, The Netherlands; 4 Department of Urology, Ng Teng Fong General Hospital, Singapore, Singapore; 5 Department of Urology, University Hospital Zurich, Zurich, Switzerland; 6 Department of Urology, AZ Klina, Brasschaat, Belgium; 7 Department of Urology and Andrology, Astana Medical University, Astana, Kazakhstan; 8 Department of Urology, Hospital Clinic, University of Barcelona, Barcelona, Spain; 9 Division of Experimental Oncology, Unit of Urology, Urological Research Institute, IRCCS San Raffaele Hospital, Milan, Italy; 10 Department of Urology, Sismanogleio Hospital, National and Kapodistrian University of Athens, Athens, Greece; 11 Department of Urology AP-HP, Tenon Hospital, Sorbonne University, Paris, France; 12 Department of Urology, Puigvert Foundation, Autonomous University of Barcelona, Barcelona, Spain; 13 Department of Urology, University Hospital Southampton, Southampton, UK

**Keywords:** Social media, urology, education, misinformation, support

## Abstract

Social media (SoMe) is now a core part of modern-day life with increased use among both patients and urologists. The interplay of SoMe between these two parties is complex. From a patient perspective, SoMe platforms can serve as educational tools as well as communication portals to support networks and patient communities. However, studies report the educational value of content online is often poor and may contain misinformation. For urologists, SoMe can lead to research collaborations, networking and educational content but areas of concern include the potential negative impact SoMe can have on mental health and sharing of patient images without appropriate consent. This review serves to provide an overview of the interaction between SoMe and urology practice and provide practical guidance to navigating it.

## Introduction

Social media (SoMe) has become a ubiquitous part of modern life. Globally, over 5 billion persons are estimated to actively use SoMe.^1^ Furthermore, individuals spend an average of 145 min per day on SoMe.^2^ It is not surprising, therefore, that its use has been extended to health-related content. This applies to the field of urology too, where an increasing number of studies have highlighted its growing presence.^3–5^ While previous studies have referred to this phenomenon as ‘emerging’, it is arguably now well established.^6^ From a perspective of patient’s and clinician’s education, there exists a clear potential for SoMe to serve as a positive resource, the opposite is also true. To this end, it has led to a situation where while certain merits of SoMe in urology can be acknowledged, there are increasing concerns regarding negative consequences such as misinformation and inappropriate conduct. The rapid growth and complex interplay between SoMe can render it difficult for the clinician to stay up to date and comprehend the key elements to be aware of. The aim of this review is to offer an examination of the relation between SoMe and the field of urology and to provide urologists with a comprehensive understanding of the nuances and implications inherent in this relationship, by elucidating how SoMe influences urology.

## Materials and methods

In this narrative review, a comprehensive search was performed for studies in the field of urology-related to social media. All article types were considered. Bibliographic databases searched included PubMed/Medline and Google Scholar. Search terms included ‘social media’, ‘urology’ and ‘SoMe’. The results were collated and presented in a narrative format with the following key areas highlighted: patient use, urologist use, impact on academic activity and areas of concern. Priority was assigned for publications within the past 10 years.

## Patient use

**Figure 1.**
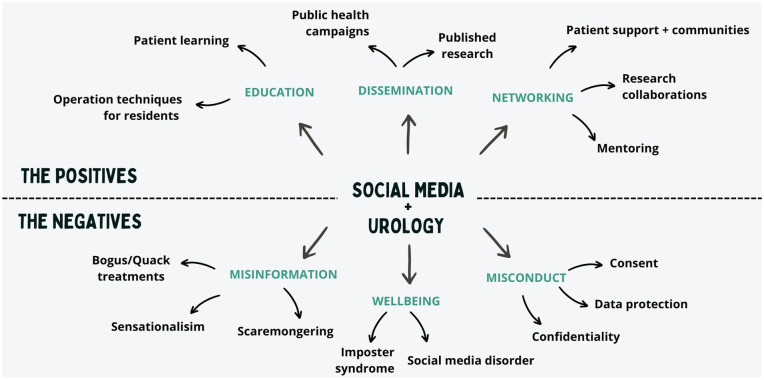
Summary of positive and negative effects of social media.

The use of the internet to seek out information related to urological conditions is high. This has been confirmed in numerous studies that have examined Google Trends in order to assess such online search behaviours.^7^ In 2019, 7% of the daily 5.5 billion Google searches worldwide were related to health.^8^ The COVID-19 pandemic, which was associated with many individuals experiencing isolation and more limited healthcare access appears to have also catalysed this shift.^9^ Younger persons and especially those with high usage of SoMe appear to use it widely as a tool for information on news and health. For example, a study of 3000 YouTube users revealed that 84.7% make decisions on their health care based on the content viewed.^10^ It is also common for patients with a particular disease to form online communities. In some formats, such as Facebook’s private/closed forums, this is more easily accomplished. Farah et al.^11^ found that for interstitial cystitis alone, there were 25 support groups on Facebook. It is consistent with the findings of Qin et al.^12^ who reported that 55.8% of patient posts on SoMe related to urogynaecology were community-building posts. Patients can therefore access a support network, which is especially valuable in cases where the disease may be rare such as in paediatrics and for parental discussion (Figure 1).^13^ SoMe can therefore act as a window for patient learning but also as a bridge for connecting with other patients.^14^ Some of these networks later develop into recognized patient advocacy groups.^15^

Using tools such as the DISCERN quality index and Patient Education Materials Assessment Tool (PEMAT), the quality of education material uploaded on SoMe has been studied across nearly all areas of urology.^16^ Much of this content is created by members of the general public, most of whom are not qualified health professionals. Regardless of the particular SoMe platform, these studies unanimously report the levels of educational content to be low. There has been an effort to create patient information videos regarding procedures by groups such as the European Association of Urology (EAU), but their viewer content [https://patients.uroweb.org/] is much lower than that of the SoMe. Engaged users are also important. The number of likes or comments can be used as a surrogate marker in this regard. It is such that with a clickbait phenomenon, posts generally get more attention if they are negative and controversial. More concerning is the high level of misinformation that is widely found. Xu et al.^17^ analysed content of prostate cancer videos on TikTok. They found significant misinformation to be present in 48% of posts. Examples included promotion of PSA screening at younger ages and offers of beverages delivering a miracle cure. Advertising and commercial posts are increasingly prevalent and account for up to 40% of all posts related to female urinary incontinence.^18^ Misinformation can lead to patients delaying and avoiding seeking medical attention. It can also potentially influence a patient’s decision to seek out experimental treatments that lack a sufficient evidence base.

Not all information that is disseminated online is erroneous and public health campaigns have had successful results. As highlighted in a systematic review by Naslund et al.^19^ tailored SoMe interventions for smoking cessation have been found to decrease the risk of relapse by between 35% and 84%.

## Urologist use

There is educational value in SoMe for clinicians. It has become a popular resource for observing surgical videos. In a survey of young urologists, YouTube was ranked higher than other websites and textbooks for learning surgical techniques.^20^ In a study analysing content related to kidney stone surgery on Instagram however, the authors found that while patients were predominantly posting content related to quality of life, this was in clear contrast to that of urologists (professional, not private accounts) who focussed on self-promotion (79%) and discussing new surgical technologies (52%).^21^ Practicing urologists should be aware that patients may film or name them individually or their hospital in their posts. In a study evaluating patients reporting experiences of kidney stone surgery on TikTok, 49% of the videos were filmed at the hospital and in 9%, the name of the hospital was clearly stated.^22^ Patients may search for their treating clinician on SoMe. A study by Koo et al.^23^ found that, of the 2015 cohort of US urology graduates (*n* = 281), 70% had an account on Facebook that was fully accessible to the public and 18% were found to include content that the authors determined to be explicitly unprofessional.

## Impact on academic activity

Hayon et al.^24^ reported that 8.9% of new articles in the field of urology are tweeted by one of the authors. Visual abstracts have been increasingly adopted to facilitate disseminating new articles. Klaassen et al.^25^ found that in a prospective trial comparing sharing either a key figure versus visual abstract, the latter resulted in higher rate of article sharing but interestingly a significantly lower rate of full link clicks. O’Kelly et al.^21^ reported that a urology journal having a social media account for sharing content was associated with a significantly higher increase in impact factor over time. Numerous studies have investigated the question as to whether sharing articles via SoMe ultimately leads to higher citation counts. Wang et al.^26^ studied the fate of 394 articles in the field of urology and recorded the tweet to additional citation rate to be very low. Rather, non-SoMe related factors had a larger influence on subsequent citations. This included prospective nature (12.9 times higher) and open access (4.3 times higher). High altimetric scores do not necessarily therefore translate to higher bibliometric scores. Indeed, a study examining the relationship between status as a urology ‘twitter influencer’ and *h*-index found the correlation to be weak.^27^

SoMe has positive implications for academic networking and scientific collaboration. Urologists also find their communities on SoMe. Many author groups have used such digital platforms to connect and form research collaboratives.^28,29^ Programmes have also been set up on SoMe to allow for residents and medical students to be paired with mentors.^30^ An example of this was the #UroStream101 initiative to support applicants in the process of US residency matching.^31^

## Areas of concern

The EAU and Americal Urological Association (AUA) have developed recommendations for appropriate SoMe use.^32,33^ However, a recent survey of 372 urology residents worldwide reported that while 99.4% use SoMe, only 34.6% had ever reviewed such material and the majority had never been given any kind of instruction on professional SoMe conduct by their institution.^34^ More worrying in this study, was the finding that 11.3% fulfilled the criteria for social media disorder (SMD), which is characterized by over usage and excess concern regarding SoMe content. SoMe can therefore have detrimental psychological effects on the individual. Popular SoMe content for urologists to view online is often surgical videos. Indeed, posting such visually appealing content (sometimes referred to under the category of ‘sensationalist’) is likely to gain the user a bigger and more quicker following. However, information regarding patient consent is often lacking if present at all. When submitting a case report to a journal, submitting authors are required to obtain formal written consent with documentation to support the patient has read and approved the content to be shared. Sharing images of cases on SoMe, seemingly misses out these obligatory steps.

A recent survey of urologist’s posts including patient related surgical or radiological images found that a clear consent statement was absent in 97% of posts evaluated.^21^ Moreover, in 3% of cases, either a name was identifiable in a radiological image, or the face was clearly visible. In cases where ethical conduct is breached, it is health directorate of the professional’s that can investigate and apply any sanctions. In 2019, 1000 persons in the UK were formally investigated for cases of misconduct on SoMe with the result of at least one clinician losing their medical licence permanently.^35^ Lawsuits can and have been successfully filed against individual doctors for breaching EU General Data Protection Regulation.

By engaging and clicking ‘like’ on such video content we see online, that may lack stringent consent measures, the urological community may therefore be unwittingly propagating such practices. Other surgical disciplines arguably face greater challenges such as plastic surgery where a patient’s face is more likely to be in the surgical field and thus filmed. In contrast, video content taken during robotic surgery in the abdominal cavity appears on the surface at least to be less problematic. Urology surgery does often involve external genitalia and this content is often visible in videos shared online. Even if a patient’s face is not identifiable, they may not be comfortable with footage of them exposed in the lithotomy position being shared freely. This also raises another issue of graphic content being online, that minors may unwittingly come across. The official minimum age for TikTok and Twitter is 13 years and no restriction for age of a follower can be applied. Urologists posting video content should also be aware they have no copyright ownership to the images.^36^ Even if a post is deleted, it can usually still be accessed on an archived site.

**Figure 2.**
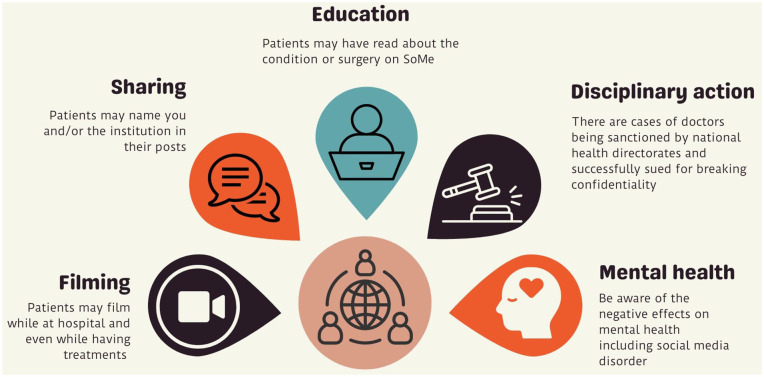
Areas of awareness for urologists in daily practice: (whether actively consuming/posting on SoMe or not).

**Figure 3.**
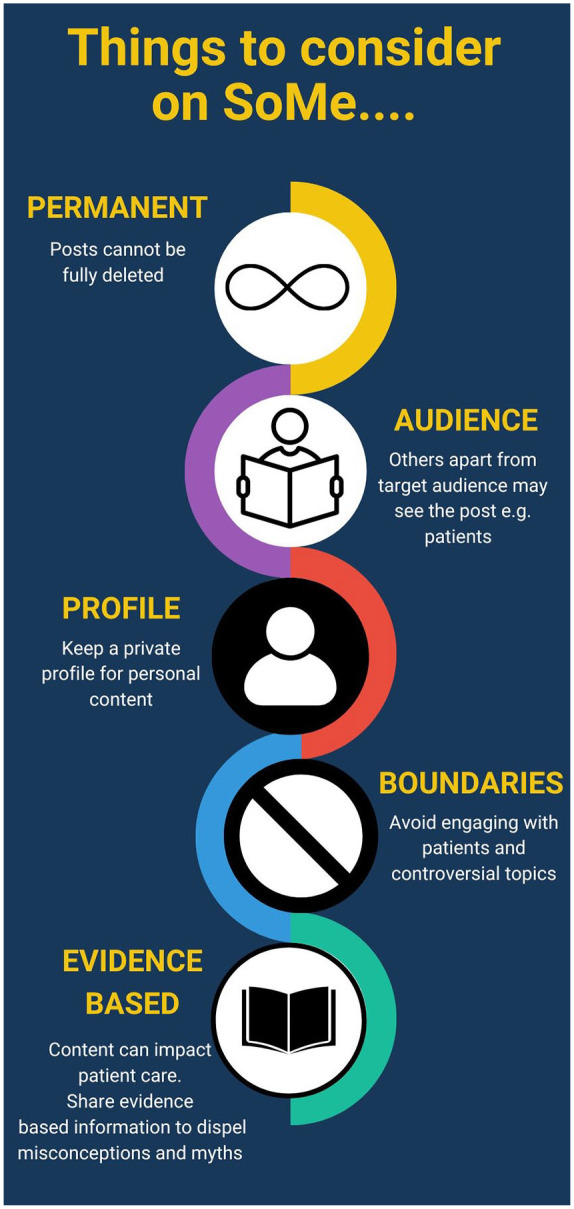
Recommendations for urologists actively posting on social media.

It Is almost impossible for a urologist to avoid the interplay of social media and urology. Indeed, with the expansion of artificial intelligence such as ChatGPT and Google Gemini, there are an increasing number of digital resources that a urologist needs to be aware of.^37^ Areas of awareness and basic recommendations that can be used are given in Figures 2 and 3.

With the widespread dissemination of medical information on SoMe, there may be a risk of patients relying more on online sources rather than seeking guidance from their healthcare providers. This shift in behaviour could lead to misunderstandings, misinterpretations, or incomplete information, affecting the quality of patient care and undermining the trust between patients and their doctors. Additionally, the influence of social media on patients’ treatment decisions and expectations, as well as the challenges faced by healthcare providers in addressing misinformation or unrealistic expectations propagated through social media, could be further explored.

## Future perspectives

Organizations such as EAU should continue to create educational content for patients while appreciating how the content creation can increase viewership and exposure to help combat posts with misinformation. Institutions are encouraged to establish formal guidance for employees. This should be disseminated internally, as well as made easily accessible.

## Conclusion

While SoMe holds the potential to enrich patient and urologist education and appears to often serve as a platform for patients to access peer to peer support, this is matched and potentially superseded by a number of pitfalls such as misinformation and breaches of patient confidentiality by health care professionals. Urologists should familiarize themselves with the current status of this interplay in order to help mitigate these risks in their clinical practice.
